# Obituary

**Published:** 1902-10-15

**Authors:** 


### OBITUARY.

### DR. ALBAN VAUGHAN ELLIOTT.

Died, at his residence, Via Tornabuoni, Florence, Italy, June 19, 1902, Alban V. Elliott, D.D.S., in his sixty-sixth year.

Dr. Elliott was born in Staten Island, N. Y., August 18, 1836. He began the study of dentistry with his brother, Dr. W. St. George Elliott, about 1880, and entered the dental department of the University of Michigan, from which institution he graduated March 23, 1881. He began the practice of his profession in London, England, in 1882. About a year later he removed to the Continent, purchasing the practice of Dr. C. M. Wright in Basle, Switzerland. A few years later he moved to Florence, Italy, where he continued until the date of his last illness.

Dr. Elliott, before his entrance upon the study of dentistry, was commissioned additional paymaster of the United States Volunteers, August 7, 1861, and accepted the appointment August 8, 1861. He was brevetted lieutenant colonel of United States Volunteers to rank from March 1865, "for faithful and meritorious services during the war," and was honorably discharged from the military service, July 15, 1866. He was a member of the Military Order of the Loyal Legion of the United States, Commandery of the State of New York, and a member of the American Dental Society of Europe. Dr. Elliott was twice married, and is survived by a widow and one son.—*Cosmos*.

### ACTION OF SAGINAW VALLEY DENTAL SOCIETY ON DEATH OF DR. J. A. WHITE.

Dr. Justus A. White, of Saginaw, was drowned during the regatta of the Little Traverse Bay Yacht Club August 11th. The boat capsized with four passengers. Three were rescued. Dr. White was 39 years of age, and was a graduate of the Chicago College ot Dental Surgery, in 1889. He came to Saginaw 15 years ago, and had built up an extensive business in dentistry, having offices in the Mason building. He was a single man, and a member of the Ten Eyck Club, residing at the club residence on North Warren avenue. He was a member of Saginaw Lodge, No. 77, F. & A. M., of Saginaw Valley Chapter, R. A. M., and St. Bernard Commandery, No. 16, K. T.

At a meeting of the dentists of Saginaw Valley, held at Dr. R. P. Alden's office, August 15, at 8 p. m., the following resolutions were unanimously adopted:

Whereas, in the death of our friend and practitioner,

Dr. J. A. White, the dentists of Saginaw Valley have sustained the loss of a beloved member, who by his dignity and councils added much to the profit and interest of the dental profession, and who did all in his power to promote the welfare and high professional character of this community; and

Whereas, we each feel the loss of a true friend, whose kindly smiles and deep experience did much to cheer and encourage; and

Whereas, we believe that the welfare of humanity through his professional attainments was ever his aim and ideal, and his every act was never to bring discredit upon the profession; therefore, be it

Resolved, that we pay such tribute to his memory as possible; that we extend our sympathy to the relatives and friends, who also have suffered loss; and further, be it

Resolved, that a copy of these resolutions be sent to his relatives, and that they by given to the local papers and professional journals for publication.

EGBERT T. LOEFFLER,
R. P. ALDEN,
EDWARD D. SLAWSON,
*Committee.*

### DR. A. WILKES SMITH.

Dr. A. Wilkes Smith, a well known dentist of Richmond, Ky., died July 27, 1902. Dr. Smith was well known in dental society gathering fifteen or twenty years ago, because he always attended the meetings and usually took some part in the proceedings. He was a man of good address and personal appearance, and of a sociable nature. He established a fine practice for himself and took great interest in the progress of dentistry as a profession in his own State. He was one of the best of the old school of practitioners, and a credit to his profession.

### DR. A. M. CALLAHAN.

Dr. A. M. Callahan, of Topeka, Kansas, died July 13, 1902. Dr. Callahan will be remembered by the class of 1875 and 76 of the Ohio Dental College. He was older than his classmates and exercised in a most affectionate way his paternal influences over the boys. Since graduating in 1875 he has had a good practice in Topeka, and although not actively engaged in the public gatherings of the profession, he has done much to maintain the reputation of the profession by doing his work faithfully. He was well thought of by his patients and the profession.

